# X Chromosome Inactivation Pattern and Pregnancy Outcome of Female Carriers of Pathogenic Heterozygous X-Linked Deletions

**DOI:** 10.3389/fgene.2021.782629

**Published:** 2021-12-17

**Authors:** Yuanyin Zhao, Jia Li, Limeng Dai, Yongyi Ma, Yun Bai, Hong Guo

**Affiliations:** ^1^ Department of Medical Genetics, College of Basic Medical Science, Army Medical University, Chongqing, China; ^2^ Department of Biochemistry and Molecular Biology, College of Basic Medical Science, Army Medical University, Chongqing, China; ^3^ Department of Gynaecology and Obstetrics, Southwest Hospital, Army Medical University, Chongqing, China; ^4^ Department of Gynaecology and Obstetrics, Xinqiao Hospital, Army Medical University, Chongqing, China

**Keywords:** heterozygous X-linked deletion, X chromosome inactivation (XCI), prenatal risk assessment, PCDH19-disorder, borjeson-forssman-lehmann syndrome

## Abstract

Prenatal risk assessment of carriers of heterozygous X-linked deletion is a big challenge due to the phenotypic modification induced by X chromosome inactivation (XCI). Herein, we described four Chinese pedigrees with maternal-inherited X-deletions above 1 Mb. The pathogenic evaluation revealed that all X-deletions are harmful to heterozygous carriers; however, the asymptomatic pregnant female carriers in these families tremendously complicate the prognostic assessment of the unborn heterozygous embryos. In this study, we detected the XCI pattern of 11 female carriers of heterozygous X-linked deletions and 4 non-carrier females in these families and performed the first prenatal XCI pattern analysis in a fetal female carrier of heterozygous *PCDH19*-deletion to make risk prediction. In an adult female who lost one copy of the terminal of X chromosome short arm (Xp), a region enriching a large number of XCI escapees, the expression level of representative XCI escape genes was also detected. Pregnancy outcomes of all families were followed up or retrospected. Our research provides clinical evidence that X-deletions above 1 Mb are indeed associated with extremely skewed XCI. The favorable skewed XCI in combination with potential compensatory upregulation of XCI escapees would protect some but not all female carriers with pathogenic X-deletion from severe clinical consequences, mainly depending on the specific genetic contents involved in the deletion region. For *PCDH19*-disorder, the XCI pattern is considered as the decisive factor of phenotype expression, of which prenatal XCI assay using uncultured amniocytes could be a practicable way for risk prediction of this disease. These results provide valuable information about the usage of XCI assay in the prenatal risk assessment of heterozygous X-linked deletions.

## Introduction

X chromosome comprises 155 million base pairs, contains upward of 1,200 genes, and is covered by a large number of repetitive sequences (Schwartz, 2013). It is well known that aberrant recombination between nearby DNA repeats is the molecular foundation of the chromosome structural abnormalities; hence, the copy number variants (CNV) on the X chromosome have a high incidence and many of them are associated with a spectrum of developmental disorders (Yuan et al., 2021). Compared to hemizygous males, female heterozygous carriers of pathogenic X-linked CNV usually present highly variable disease penetrance and phenotype expression, making a great difficulty for their prognostic evaluation, a leading cause of which is the phenotypic modification effect of X chromosome inactivation (XCI) (Puck and Willard, 1998; Brown and Robinson, 2000; Lyon, 2002).

XCI is an epigenetic process to equalize the dosage of X-linked genes between XX females and XY males through transcriptionally silencing one of the two X chromosomes in somatic cells of the mammalian female during early embryogenesis (Lyon, 1961). In most females, XCI is a random process. Parental X chromosome has the same probability to be inactivated in each cell at the initial time and then remains inactive in all descendant cells, ultimately giving rise to mosaicism of two roughly equal populations of cells expressing either the paternal or the maternal X chromosome (Lyon, 1961). However, sometimes the XCI is non-random, leading to a cell ratio significantly deviating from 1:1, which is called non-random (skewed) XCI (Minks et al., 2008). Generally, non-random XCI can arise through situations as below: (i) non-random XCI arising by chance, which refers to the stochastic statistical distribution of inactivation in a relatively small cell number when XCI takes place (Minks et al., 2008); (ii) primary non-random XCI, which is a rare condition that refers to the preferential choice of which X chromosome (paternal or maternal) is inactivated at the initial stage. This condition usually results from disruption of the key genomic elements that coordinate the XCI process (Rastan, 1982; Gendrel and Heard, 2011); (iii) secondary/selective non-random XCI, which refers to skewed XCI that takes place during the clonal propagation process of the post-inactivation cells rather than the initial “chromosome-choice” stage. In this situation, initial inactivation of the parental X chromosome is random, while descendant cells expressing one of the X chromosomes would be privileged in their cell growth and/or survival during subsequent mitosis, thus gradually establishing a skewed cell proportion in somatic cells (Gendrel and Heard, 2011). Secondary non-random XCI is most common and always linked to a carrier status of certain deleterious X-linked mutations including unbalanced structural abnormalities (deletions, duplications, and isochromosomes) (Leppig and Disteche, 2001). Cells containing such X chromosomes are always considered to lose growth and/or survival privilege and would be gradually eliminated with continuous mitosis. Thus, unbalanced abnormalities on the X chromosome are assumed to have a less phenotypic impact than that of similar size involving autosomes (Orstavik, 2009).

The epigenetic inactivation can spread the entire X chromosome, but not all X-linked genes are subject to the transcriptional silence. Genes that continue to be expressed from inactivated X are defined as XCI escape genes/escapees. It is estimated that at least 23% of X chromosome genes escape the XCI to some degree (Tukiainen et al., 2017), bi-allelic expression of which is essential to normal female development. Furthermore, XCI escapees are not distributed randomly along the X chromosome; many more are located on the distal short arm of the human X chromosome-Xp (21%) than on the long arm-Xq (3%) (Disteche, 1999). This finding provides an important implication for genetic counseling that structural imbalance on Xp may have greater clinical significance than that on Xq, where the effect could be largely mitigated by XCI.

Although X chromosomes with unbalanced structural abnormalities are believed to be selectively inactivated, XCI pattern analysis is rarely performed in this kind of clinical case. In this study, we collected four pedigrees carrying maternal-inherited pathogenic X-linked deletions above 1 Mb. We analyzed the XCI pattern of peripheral blood lymphocytes in all female carriers and several non-carrier females in these families, conducted the first prenatal XCI assay in a fetal heterozygous carrier of the whole *PCDH19*-deletion using uncultured amniocytes, detected the expression level of representative XCI escape genes in an adult female carrier of Xp-terminal deletion, and followed up or retrospected pregnancy outcomes of all female carriers. Our experiences provide useful references for prenatal risk assessment of heterozygous X-linked deletions and laid a foundation for further study of prognostic valuation of XCI pattern for these cases.

## Materials and Methods

### Study Participants

**FIGURE 1:**
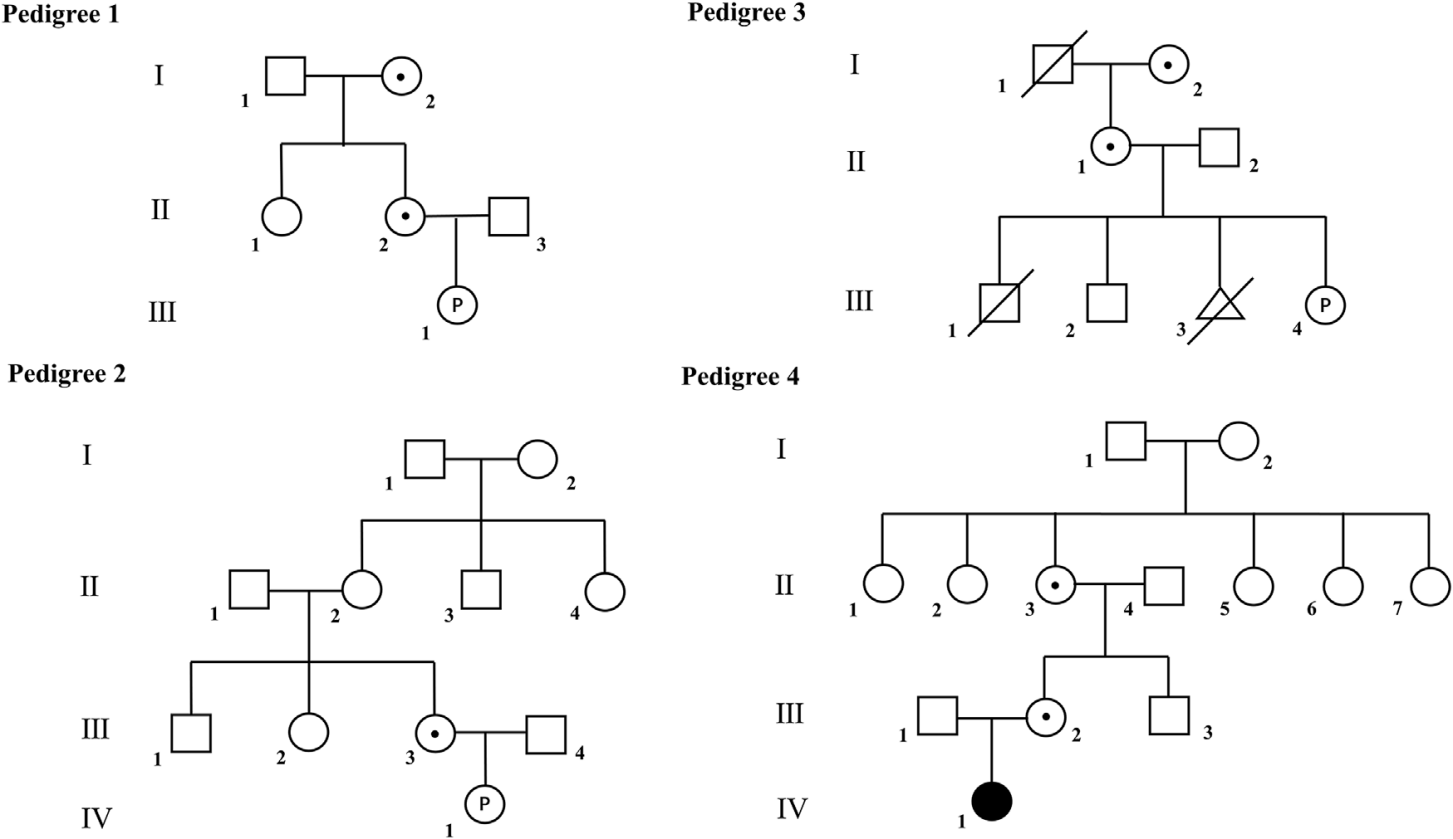
Pedigrees of the families recruited in this study. Filled symbol indicates affected individuals; open symbols indicate unaffected individuals; open symbols with dot indicate asymptomatic carriers. Open symbols with letter “P” indicate the unborn fetus.

This descriptive study focused on four independent Chinese pedigrees who underwent pre- or post-natal diagnosis (Figure 1). All pregnant women in the study got amniocentesis for chromosome micro-array (CMA) testing due to risky indications of non-invasive prenatal test (NIPT). The indication for a postnatal genetic test of pedigree four is a birth defect history in this family. Genetic tests revealed that all pedigrees contain more than one female carrier of heterozygous X-linked deletions. The common characteristic of these X-deletions is that the deletion region is above 1 Mb and contains at least one identified disease-causing gene that primarily affects heterozygous females. The study was approved by the Ethical Committee of Xinqiao Hospital, Chongqing, China.

### Molecular Diagnosis and Variant Classification

The pregnant women in this study underwent NIPT at 14–18 weeks of gestation for routine prenatal examination. The procedure was performed using the Ion Proton Sequencer (Life Technologies) according to the reported document (Yin et al., 2015). Amniocentesis was performed before 24 weeks of gestation following the ISUOG practice guidelines (Ghi et al., 2016) for indication of NIPT. Subsequent CMA using Affymetrix CytoScan HD chips was performed to detect fetal chromosome abnormality. Amniotic fluid fetal DNA was processed by sequential steps of digestion, polymerase chain reaction (PCR), PCR-product check, purification, quantization, fragmentation, and QC gel labeling, hybridization, washing, staining, and scanning according to the manufacturer’s instruction. The data were analyzed using Affymetrix Chromosome Analysis Suite Software (version4.0). The interpretation of CNVs follows the guideline recommended by the American College of Medical Genomics (ACMG) and the Clinical Genome Resource (ClinGen) (Riggs et al., 2020).

Quantitative real-time PCR (Q-PCR) and peripheral blood karyotyping were used to pedigree verification. X-deletions below 5 Mb taken by pedigree 1 and 2, respectively, were confirmed by Q-PCR. In each case, three pairs of primer were designed to amplify the distinct coding regions of the deletion interval, respectively (Supplementary Figure S1). Standard 2-step-Q-PCR was performed using IQ^TM^ SYBR Green Supermix (Bio-Rad, Hercules, CA, United States) on the CFX Connect Real-time PCR Detection System (Bio-Rad, Hercules, CA, United States). Each 20 µl assay was performed in triplicate and contained 20 ng of gDNA, 800 nM each of forward and reverse primers for the reference gene (RNaseP) or for the target genes. Relative quantity is determined by the 2^-∆∆Ct^ method (Livak and Schmittgen, 2001), where a calibrator sample known to have two copies of the test sequence is used as the basis for comparative results. Gene copy number is 2 × the relative quantity. X-deletions above 10 Mb found in pedigree 3 and four were verified using karyotyping. Approximately 2 ml of peripheral blood of the fetus’s parents and grandparents were collected to carry out G-banding analysis at a resolution of approximately 320–400 bands following standard laboratory protocols and ISCN 2016.

Trio-whole exome sequencing (WES) was carried out in pedigree 4. Exome targets were captured using the Agilent Inherited Disease Panel (Agilent Technologies, Inc.) and sequenced on the HiSeq 2,500 platform (Illumina, Inc.). Read alignment against the human reference genome builds hg19 was performed using the software NextGENe. Variants present at allele frequency greater than 0.5% in genomic databases (dbSNP138, 1,000 Genomes, ExAC) were disregarded. The software ANNOVAR was used to annotate variants. All relevant variants were visualized with Integrative Genomics Viewer (IGV) to exclude false results.

### X Chromosome Inactivation (XCI) Assay

The 5meCpG-based RP2/AR repeat biplex assay, a PCR assay that detects 5meCpG statuses of short tandem repeats (STR) alleles on the Xp (RP2) and the Xq (AR) in a single reaction, was employed to determine the XCI pattern (Machado et al., 2014). For each sample, 500 ng of gDNA was digested overnight with methylation-sensitive *Hp*aⅡ restriction endonuclease (New England Biolabs, Ipswich, MA) at 37°C. Digested products together with mock-digested products (approximately 50 ng/sample) were used as templates for quantitative fluorescence polymerase chain biplex reactions (QF-PCR). PCR products were separated *via* capillary electrophoresis on an ABI 3130XL genetic analyzer (Applied Biosystems, Foster City, CA, United States). Allele profile and peak areas were analyzed by GeneMapper software (Applied Biosystems, Foster City, CA, United States). The informative allelic statuses were used to calculate the X chromosome inactivation ratio according to a previously published method (Jones, 2014). Interpretation of XCI pattern for a given sample was as follows: random (below 80:20), moderately skewed (80:20 to 90:10), and extremely skewed (above 90:10).

### Gene Expression Assay

Given that the deletion region taken by pedigree 3 contains a large number of clinical relative XCI escape genes (Tukiainen et al., 2017), the XCI pattern alone is insufficient to explain the phenotype expression of the carriers, and the expression level of these genes is indispensable for comprehensive risk assessment for this family. Therefore, total RNA of peripheral blood lymphocytes from II-1 of pedigree 3 and four control peoples was extracted and reverse transcribed into cDNA to compare the expression level of representative genes that escape X-inactivation in pseudoautosomal region 1. Expression assay based on quantitative PCR was performed using IQ^TM^ SYBR Green Supermix (Bio-Rad, Hercules, CA, United States) on the iQ-5 Real-time PCR Detection System (Bio-Rad, Hercules, CA, United States) as mentioned above. The expression level of a tested gene was normalized to β-actin mRNA. The relative level of mRNA was determined using the 2^−∆∆Ct^ method (Livak and Schmittgen, 2001). Results are displayed as the mean ± SD from triplicate samples for each group. Significant differences were established by the one-way ANOVA followed by the Tukey-Kramer multiple comparisons post-test using the computer program GraphPad Prism (GraphPad Software Inc. V5.0, San Diego, CA, United States).

### Genetic Counselling and Follow-Up After Birth

All tested family members were told the results of molecular diagnosis and XCI pattern assay. The variant classification and the potential consequences for the fetal carriers or recurrency risk of next pregnancy have been clearly described. Women who choose to continue pregnancy were contacted by telephone to inquire their pregnancy outcomes.

## Results

### Clinical Presentation and Molecular Diagnosis

#### Pedigree 1

A 24-year-old pregnant woman (II-2), G1P0, referred for 15q11.2-15q13.1 deletion of the fetus (III-1) was indicated by NIPT. Amniocentesis followed by CMA preclude the suspected microdeletion on chr15 but uncovered a 3.557 Mb heterozygous deletion in the Xq21.33-Xq22.1 region of the X chromosome (chrX: 96821302-100378384, hg19), which involves a total of 11 OMIM genes (Table 1). Among them, *PCDH19* gene (MIM: 300460) has been documented as a haploinsufficiency gene (HI gene) in the ClinGene database. Loss-of-function of *PCDH19* can result in a rare early infantile epileptic encephalopathy (MIM: 300088, EIEE9), which almost exclusively occurs in heterozygous females. In the DECIPHER database, this interval embraces a likely-pathogenic document without phenotype description (ID:300247). There was no evidence to refuse pathogenicity in the Database of Genomic Variants (DGV Gold standard variants). Taken together, this X-linked deletion should be assigned to pathogenic CNV. However, following pedigree analysis (summarized in Supplementary Table) demonstrating that this pathogenic CNV inherited from the fetus’s apparently healthy mother (II-2), both the pregnant woman and her mother (I-2) are heterozygous carriers of the same X-deletion (verified by Q-PCR and shown in Supplementary Figure S1).

**TABLE 1:** Summary of the X-linked deletions carried by four pedigrees.

Pedigree number	Deletion position (hg19) and size	Primary disease-causing genes and related diseases	Inheritance pattern	Pathogenic curation
1	chrX:96821302-100378384, Xq21.33-Xq22.1 3.55 Mb	*PCDH19* (*300460) related early infantile epileptic encephalopathy (EIEE9, #300088)	Maternal	Pathogenic
2	chrX:47249368-50896523, Xp11.23-Xp11.22 3.65 Mb	*PORCN* (*300651) related focal dermal hypoplasia (#305600)	Maternal	Pathogenic
		*EBP* (*300205) related X-linked dominant chondrodysplasia (#302960)		
		*SLC35A2* (*314375)-related congenital disorder of glycosylation, type II m (#300896)		
		*WDR45* (*300526)-related neurodegeneration with brain iron accumulation 5 (#300894)		
3	chrX:178624-18404079, Xp22.33-Xp22.13 18.225 Mb	*SHOX* (*312865) related Leri-Weill dyschondrostosis (LWD, #127300)	Maternal	Pathogenic
		*HCCS* (*300056) related linear skin defects with multiple congenital anomalies1 (#309801)		
		*OFD1* (*300170) related orofaciodigital syndrome1 (#311200)		
		*NHS* (*300457) related Nance-Horan syndrome (#302350)		
4	chrX:129511205-142742928, Xq26.1-Xq27.3 13.23 Mb	*F9* (*300746) related hemophilia B (#306900)	Maternal	Pathogenic
		*FRMD7* (*300628) related nystagmus-1 (#310700)		
		*GPC3*(*300037) related Simpson-Golabi-Behmel syndrome, type 1 (#312870)		
		*HPRT1*(*308000) related Lesch-Nyhan syndrome (#300322)		
		*PHF6*(*300414) related Borjeson-Forssman-Lehmann syndrome (#301900)		
		*SLC9A6* (*300231) related Christianson type of X-linked syndromic mental retardation (#300243)		

Detailed genomic locations and contents (the haploinsufficiency genes primarily affecting female carriers) of the four distinct X-linked deletions were listed in the table. The inheritance patterns of these X-linked deletions were determined by the pedigree analysis presented in supplementary table. The pathogenic curation was made according to ACMG guideline (Riggs et al., 2020).

#### Pedigree 2

A 28-year-old pregnant woman (III-3), G1P0, underwent amniocentesis because NIPT indicated a 4.00 Mb deletion in the Xp11.23-Xp11.22 region of the fetus’s X chromosome. Subsequent CMA performed on amniocytes confirmed that the fetus (IV-1) carried a heterozygous deletion of chrX: 47249368-50896523 (hg19) region. In DGV database, no CNV including this deletion has been recorded. In DECIPHER database, this region contains a pathogenic report described as a heterozygous carrier with intellectual disability (ID:322311). Assessment of genomic contents uncovered that this interval contains 72 OMIM genes, 7 of which have been curated haploinsufficiency. Among the 7 HI genes, 4 genes are relative to X-linked dominant diseases that primarily affect heterozygous females (summarized in Table 1). In this case, we already have enough compelling evidence to call this CNV pathogenic. However, evaluation of the inheritance pattern revealed that the deleted allele was inherited from the fetus’s mother (demonstrated by CMA and Q-PCR) and the microdeletion is a *de novo* mutation for the apparently unaffected pregnant woman (III-3) because the Q-PCR verification showed that the pregnant woman’s parents, sister, and maternal aunt do not carry the deletion (detailed information was shown in Supplementary Table and Supplementary Figure S1).

#### Pedigree 3

A pregnant woman of 33 years old (II-1), G4P3, gave birth to two healthy boys in 2012 and 2015, respectively (the elder brother died in an accident) and terminated the third pregnancy for personal reasons in 2017. In the fourth pregnancy, NIPT and CMA uncovered an 18.225 Mb-heterozygous deletion spanning Xp22.33-Xp22.13 (chrX: 178624-18404079, hg19). There is no record that completely overlapped this interval in the population database. In DECIPHER, the region involves 2 types of syndromes: Leri-Weill dyschondrostosis (LWD) and Steroid sulphatase deficiency and numerous unique loss-pathogenic documents. The clinical phenotypes of these heterozygous cases involved short stature (ID:307967, 363886, 391541), autism and language development disorders (ID:338884), and global development delay (ID: 285776, 285002, 301496). In this region, 11 curated haploinsufficiency genes were covered, 4 of which primarily affect heterozygous women (summarized in Table 1). All of the evidence listed above are enough to demonstrate the pathogenic nature of the CNV. However, just like pedigree 1 and 2, the mutant allele is also maternally inherited. Karyotyping displayed that the mother (II-1) and the maternal grandmother (I-2) of the fetus present the same abnormal karyotype: 46, X,del (X) (p22.1) (summarized in Supplementary Table), but no severe manifestation was presented by the two carriers besides isolated short stature (both two carriers’ height was 150 cm, −1.9 SDS; other features of LWD, such as limb shortening or abnormality of the forearms, were absent).

#### Pedigree 4

A 30-year-old woman (III-2) asked for pregnancy counseling because she gave birth to a girl (IV-1) presented with global developmental delay and atrial septal defect in 2014. CMA performed on the girl uncovered a deleterious copy number variant: Xq26.1-q27.3 (129511205-142742928, hg19) × 1. The 13.2 Mb-deletion region contains 62 OMIM genes, 8 of them have been curated as HI genes (partial female-affected HI genes are summarized in Table 1). Although a vast majority of potential diseases caused by deletion of this region primarily affect hemizygous males, affected cases have been observed in heterozygous carriers of HI genes mentioned above. Actually, several loss-pathogenic reports involving heterozygous females in this region have been documented in DECIPHER (ID: 424282 and 318740 mainly involving *GPC3* gene; ID: 262468 and 402512 mainly involving *PHF6* gene). Whole or partial deletion of the *PHF6* gene (MIM: 300414) leads to an X-linked disorder called Borjeson-Forssman-Lehmann syndrome (BFLS, MIM: 301900), which usually affects males, but mild to severe symptoms are also present in female carriers. In our case, phenotypic features of the patient highly overlapped with the clinical presentation of previously reported female BFLS patients (Zweier et al., 2014), which includes developmental delay with severely affected speech and symbolic physical features (sparse hair, tapering, and fifth curved fingers/toes, skin hyperpigmentation, and narrow auditory canal, presented in Supplementary Figure S2). Subsequent pedigree investigation (summarized in Supplementary Table) uncovered that the unaffected mother (III-2) contains the same CNV as her sick daughter (indicated by CMA). The G-banding analysis performed on other family members (Supplementary Table) displayed that the maternal grandmother (II-3, unaffected) of the patient owns an abnormal karyotype: 46,X,de l(X) (q24q26). Trio-WES (including III-1, III-2, and IV-1) was also involved to preclude other potential disease-related single nucleotide variants or indels; no suspected variants could well explain the phenotypes of the patient. Taken together, we concluded that the deleterious allele causing BFLS is maternally inherited.

### X Chromosome Inactivation (XCI) Assay

Given XCI is a vital factor to modify the clinical consequences of female carriers of X-linked disorder, the XCI pattern of all female carriers of X-linked deletion and four non-carrier females in these pedigrees was detected using blood lymphocytes. Our results (Table 2) displayed that all female carriers present a completely skewed XCI pattern (100:0), while random XCI occurred in all tested non-carrier females. Among the female carriers with favorable XCI patterns against deleted alleles, the five-year-old girl with BFLS syndrome (in pedigree 4) was the only one who has obvious clinical manifestations. In pedigree 4, heterozygous carriers of Xq26.1-q27.3 deletion in successive three generations exhibited the same XCI pattern (extremely skewed XCI pattern −100% towards null allele, shown in Table 2) but completely different phenotypes: the mother (III-2) and the maternal grandmother II-3) are asymptomatic, while the proband present obvious manifestations of BFLS. This phenomenon implied that the XCI pattern of blood cell could not be a reliable indicator of the clinical consequences of this disease.

**TABLE 2:** The XCI patterns of the female relatives in four pedigrees.

Pedigree	Family member	Blood DNA XCI ratio		Most inactive chromosome	Origin of X-linked deletion
		AR alleles	RP2 alleles		
1	I-2^‡^	n/i	100:0	n/p	n/p
	II-2^‡^	100:0	n/i	Maternal	Maternal
	III-1^‡^	100:0	n/i	Maternal	Maternal
2	II-2	79:21	67:33	—	—
	II-4	69:31	63:37	—	—
	III-2	n/i	51:48	—	—
	III-3^‡^	100:0	100:0	Paternal	*De novo*
	IV-1^‡^	n/i	100:0	Maternal	Maternal
3	I-2^‡^	100:0	100:0	n/p	n/p
	II-1^‡^	100:0	100:0	Maternal	Maternal
	III-4^‡^	100:0	100:0	Maternal	Maternal
4	II-1	n/i	77:23	—	—
	II-3^‡^	n/i	100:0	n/p	n/p
	III-2^‡^	100:0	100:0	Maternal	Maternal
	IV-1^‡^	100:0	n/i	Maternal	Maternal

X chromosome inactivation (XCI) assay based on 5meCpG statuses of STR alleles on the Xp (RP2) and the Xq (AR) were performed in 11 heterozygous carriers of X-linked deletions (indicated by symbol ‡ in column 2) and four non-carrier females in four pedigrees. The informative allelic statuses (n/i: non-informative allele) were used to calculate the X chromosome inactivation ratio (described in “materials and methods”). The XCI ratio below 80:20 is interpreted into a “random inactivation pattern”, while the XCI ratio above 90:10 means “extremely skewed inactivation”. The most inactivated X chromosome of a given sample was determined by trio-STR genotyping (n/p: the trio-STR genotyping is not performed). The origin of X-deletion of a given sample was determined by Q-PCR or karyotyping presented in supplementary table.

**FIGURE 2:**
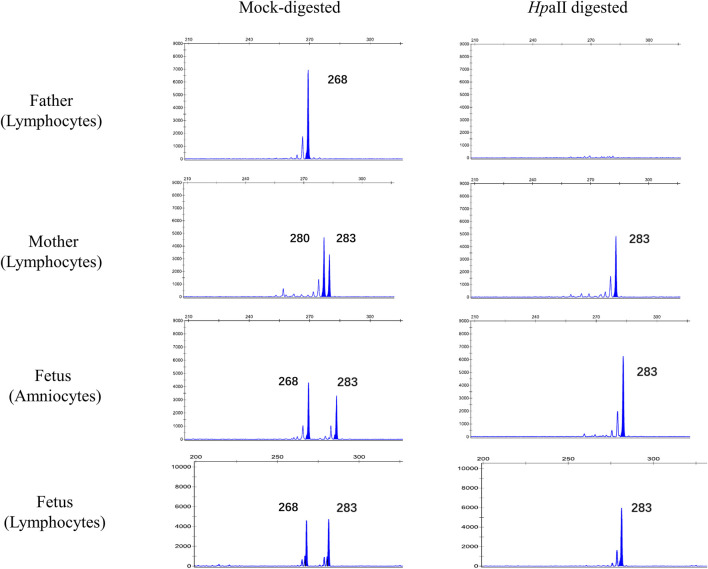
Prenatal XCI assay performed in the female fetus of pedigree 1. gDNA extracted from fresh uncultured amniocytes of the fetus and peripheral blood lymphocytes of her parents was used to detect 5meCpG statuses of *AR* and *RP2* alleles. The peaks indicate the informative *AR* alleles. The size of each allele is determined by the length of polymorphic microsatellite in the exon1 of *AR* gene. The allele^268^ and allele^283^ of the fetus is inherited from her father and mother, respectively. After digestion by methylation-sensitive HpaⅡ restriction endonuclease, bi-allelic status of the mother and fetus’s AR gene converts to a mono-allelic status, which means that both of them possess completely skewed XCI pattern and the remaining alleles represent the alleles that have been inactivated (methylated). Thus, for the fetus, skewed XCI towards allele^283^ of her, which indicates the maternal-original X chromosome (the one taking microdeletion), has been inactivated. The XCI pattern of amniocytes is perfectly replicated in neonatal cord blood lymphocytes.

Moreover, in pedigree 1, which contains a fetus having a risk of *PCDH19*-deletion disorder, we detected the fetus’s XCI pattern in prenatal using uncultured amniocytes. The result revealed that the female fetus presents a 100% XCI skewing against the maternal (origin of deletion) X-chromosome, which implies a positive prognosis. Furthermore, the XCI pattern of amniocytes was consistent with that of neonatal cord blood lymphocytes detected after birth (Figure 2), indicating the feasibility of XCI assay in amniocytes.

### The Expression Level of XCI Escapees Located in Deletion Region of Pedigree 3

**FIGURE 3:**
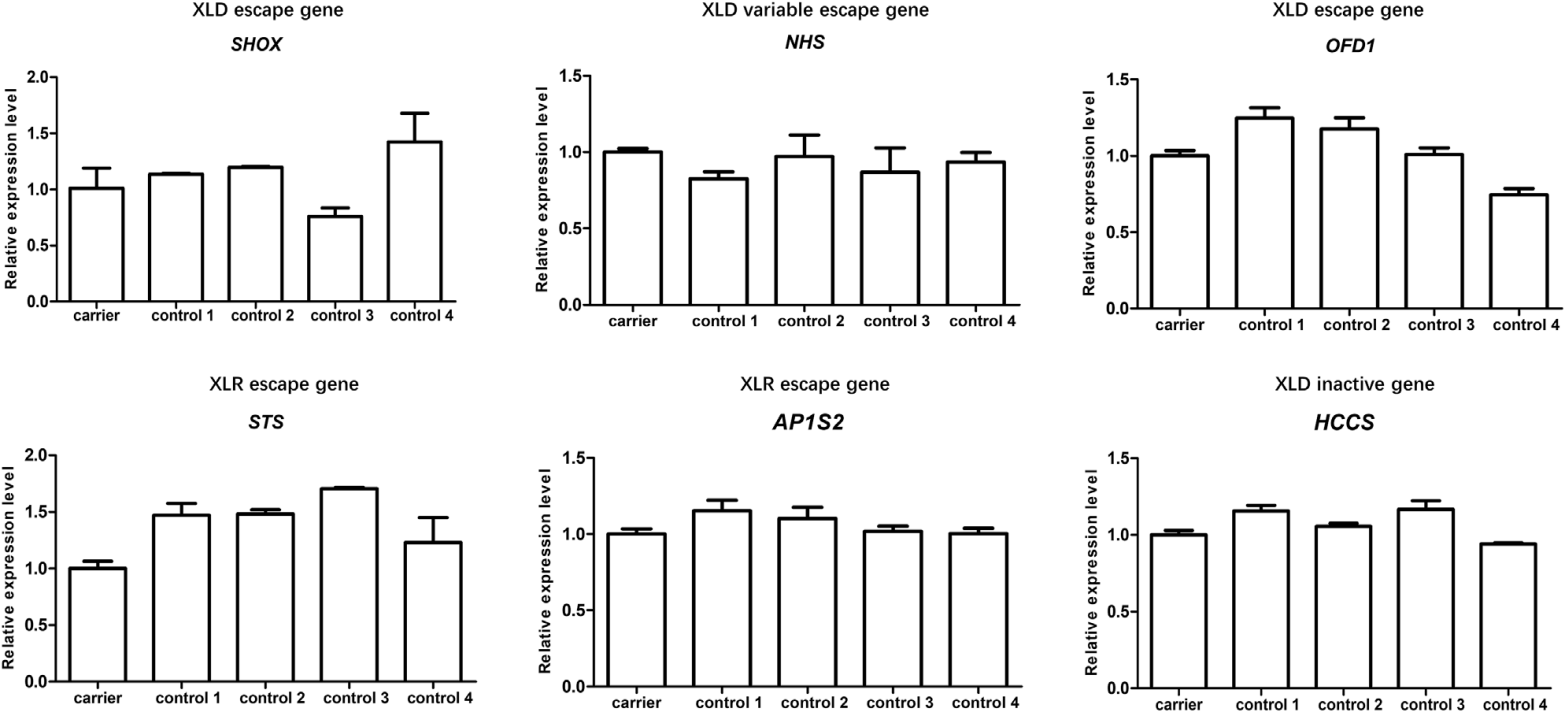
The expression levels of representative genes involved in the deletion region of pedigree 3. Total RNA was extracted from peripheral blood lymphocytes of one female carrier of Xp22.33-Xp22.13 deletion (II-1in of pedigree 3) and four race- and age-matched non-carrier female controls. qRT-PCR was used to detect the expression of six representative genes located in the deletion region in these samples. The inheritance mode and reported XCI status of these gene have been annotated in the figure. The relative levels of these genes are normalized to β-actin expression. Data represents the mean of experiment performed in triplicate; there is no significant difference among the tested samples.

It is known that the majority of escapees of XCI are located on the Xp, especially highly enriched in the pseudoautosomal region 1 (PAR1). The 18.225 Mb deletion taken by pedigree 3 contains whole PAR1 and adjacent escapee strata, including 44 defined XCI escape genes (Tukiainen et al., 2017), which account for half of the total number of genes in this region. Theoretically, irrespective of the deletions of escape genes that occurred in the active X chromosome (Xa) or inactive X chromosome (Xi), they could be harmful. However, our study revealed that in comparison to four race, age, and sex-matched controls, the copy number alteration at the genomic level of several potential disease-causing escape genes in our female carrier did not result in significant dosage insufficiency at the transcriptional level (Figure 3), which implied that potential upregulation of escapees from intact X chromosome could equalize the gene dosage between bi-allelic female and structurally mono-allelic female.

### Genetic Counseling and Clinical Outcome Follow-Ups

For the pedigrees with fetal carriers of X-deletions, comprehensive prognostic evaluation was informed to the pregnant women. After a full consultation, all of them decided to continue their pregnancies. Follow-up so far showed that the baby girl with a risk of EIEE9 in pedigree 1 has not suffered from seizures during the 16 months after birth (the average onset age of EIEE9 is 14 months (Samanta, 2020)); the baby girl harboring a heterozygous deletion related to multisystem anomalies and intellectual disability (in pedigree 2) has no other distinguishing features at 10 months after birth, aside from many café au lait spots distributed to the whole body (a retrospective survey found that similar pigmentation of skin also occurred in her “non-phenotypic” carrier mother); the baby girl (in pedigree 3) with deletion of numerous XCI escapees including *SHOX* presented with normal growth milestones and limb length during the first year after birth.

We assessed the recurrence risk for the couples (III-1, III-2 in pedigree 4) who gave birth to a BFLS girl. In their every pregnancy, there would be a 50% chance to transmit the deleted-*PHF6* allele to their offspring, irrespective of the sex. Males who inherited the deleted allele would 100% present full-blown BFLS phenotypes, and even for female heterozygous, the penetrance is estimated up to 44% (Zhang et al., 2019). More importantly, our study and quite a bit of previous cases showed that favorable skewed XCI is usually insufficient to prevent harmful outcomes for this disease (Crawford et al., 2006; Berland et al., 2011; Zhang et al., 2019). After careful consideration, the couples chose preimplantation genetic diagnosis (PGD) to prevent the transmission of the pathogenic CNV.

## Discussion

In this study, we reported four pedigrees carrying different heterozygous X-linked deletions (>1 Mb), which were discovered incidentally through prenatal screening (their unborn fetuses were indicated as X chromosome deletion by NIPT) or diagnosed during the retrospection of a birth defect history (pedigree 4). According to the ACMG interpretation framework (Riggs et al., 2020), all microdeletions should be assigned to pathogenic CNV for heterozygous carriers. However, the fact that these CNV are inherited from the unaffected mothers complicates the valuation of their clinical implications. Given several X-linked disorders including X-deletion have been reported to be associated with skewed XCI and normal phenotype in heterozygous carriers (Orstavik, 2009), we set out to determine the XCI pattern of female relatives in our pedigrees at first. We found that a total of 11 X-deletion female carriers 100% presented completely skewed XCI pattern against abnormal X-chromosome, while the prevalence of XCI skewing in non-carrier females is zero (4/4 random XCI), which seems highly consistent with the principle that the unbalanced structurally abnormal X chromosome is preferential to be inactivated. However, limited by the small number and types of cases in our study, a definitive conclusion about the relationship between X-structural abnormality and XCI pattern cannot be drawn just based on this observation. For instance, our study did not show whether the X-microdeletion below 1 Mb would also lead to selective XCI skewing and we also noticed that certain X-linked CNV (such as Xp11.23-p11.22 duplication) exhibits an unusual skewed XCI pattern that is in favor of the aberrant X chromosome (Di-Battista et al., 2016). Therefore, we proposed that the skewed XCI associated with X chromosome structural abnormality highly depends on the size and particular genomic contents of the variant region. Systemic investigations focused on the XCI patterns in various X chromosome structural anomalies will make a better understanding of the correlation between them.

More importantly, even though the favorable skewed XCI against abnormal X chromosome occurred, does that definitely indicate a positive prognosis? Our experiences suggest that the prognostic value of the XCI pattern is inconsistent in disorders with different genetic foundations. For example, in our cases, skewed XCI seems a positive prognostic indicator of *PCDH19*-deletion (3 asymptomatic heterozygous carriers of *PCDH19*-deletion with completely skewed XCI in pedigree 1), due to the special “mosaic pathogenesis” of PC*DH19*-disorder (Dibbens et al., 2011; Dimova et al., 2012; Terracciano et al., 2012; Hoshina et al., 2021). *PCDH19* gene encodes a single transmembrane protein involved in calcium-dependent cell-cell interaction and adhesion. Cellular interference between wild type-*PCDH19* neurons and mutant/deleted-*PCDH19* neurons is the main pathogenic mechanism underlying the disorder (Hoshina et al., 2021). Therefore, the heterozygous females with extremely skewed XCI always remain spared due to a low level of somatic mosaicism of this gene. However, for some other X-linked disorders, such as BFLS induced by *PHF6* gene mutation, our case (in pedigree 4) and numerous previous reports all uncovered that the XCI pattern of blood cell has poor prognostic value for clinical consequences (Crawford et al., 2006; Berland et al., 2011; Zhang et al., 2019). The reasonable explanation could be that unlike *PCDH19*, expression of which peaks in the first postnatal period (Hoshina et al., 2021), *PHF6* is primarily expressed in an early stage of fetal brain development (Jahani-Asl et al., 2016). At that time, the concrete ratio and distribution of the two populations of cells (expressing normal or mutant *PHF6*, respectively) are stochastic and almost impossible to be accurately reflected by an XCI ratio established in peripheral blood cells after birth because the XCI ratios of tissues in different development stages are inconsistent. Generally, the more mitosis experienced, the more skewed the XCI ratio presented due to clonal selection (Minks et al., 2008). Therefore, employing the XCI assay in the risk prediction of certain X-linked disorders must take into account the particular pathogenic mechanism of the disease and the representativity of the alternative tested tissues.

When we discuss the relationship between XCI and phenotypes of X-linked disorder, we cannot ignore the presence of a considerable number of XCI escapees. In pedigree 3, a three-generation family takes a heterozygous deletion located in the most enriched region of XCI escapees (Tukiainen et al., 2017). This “escapee cluster” encompasses several X-linked dominant (XLD) escape genes either with high penetrance (such as *SHOX*, MIM: 312865, haploinsufficiency of which resulted in short stature in varying degree) or with severe phenotypes (such as *OFD1*, MIM: 311200, mutation of which could be lethal in males). Theoretically, regardless of the XCI pattern, the dosage imbalance of these important XLD escape genes at the genomic level would result in severe phenotypes. Surprisingly, there are no significant clinical symptoms presented in our carriers. Karyotyping has ruled out post-zygotic mosaicism involving the concomitant presence of normal and X-deletion cells; thus, we compared the expression level of six representative genes involved in the deletion between one of our carriers (II-1 of pedigree 3) and four race- and age-matched non-carrier females. The nearly equalized expression of all XCI escapees among them exactly coincides with a scenario reported by Santos-Reboucas et al. recently, who observed a skewed XCI and compensatory upregulation of escape genes in an asymptomatic female with a *de novo* heterozygous deletion (32 Mb) containing Xq-escapee cluster (Santos-Reboucas et al., 2020). Both two cases suggested that large deletion of the X chromosome could induce a potential dosage compensation mechanism to balance the expression of XCI escapees between bi-allelic females and structurally hemizygous females. However, due to few related cases having been reported so far, the universality and molecular mechanism of the dosage compensation effect merit further investigation.

As we mentioned above, the XCI pattern is significantly implicated in the phenotype expression of *PCDH19*-disorder. Nevertheless, the clinical application of XCI assay in the risk prediction of the disease has not been reported. The free cells from amniotic fluid contain multi-organ cells of fetal, such as skin, gastrointestinal, and urinary tract cells, in theory, should be more appropriate for XCI assay than most used blood cells. Moreover, it has been demonstrated that the XCI pattern of amniocytes has been established by the time of routine prenatal diagnosis and used to predict the risk of dystrophinopathy in prenatal (He et al., 2019). Therefore, in this study, we performed the first prenatal XCI assay using uncultured amniocytes to evaluate the prognosis of a fetal heterozygous carrier of *PCDH19-*deletion. Although the validity of this analysis is needed to be assessed by further studies with a large number of cases, our study makes the first step towards the prenatal risk prediction of *PCDH19*-disorder using the XCI pattern.

In conclusion, the female carriers with heterozygous X-linked deletion (>1 Mb) would undergo XCI skewing. The favorable skewed XCI in combination with potential compensatory upregulation of XCI escapees would protect a part of female carriers with pathogenic X-deletion from severe clinical consequences. The prognostic value of the XCI pattern depends on particular genomic contents of the deletion region and the choice of appropriate tested tissue. For certain diseases like loss-of-function of the *PCDH19* gene, the XCI pattern could be a useful prognostic indicator. XCI analysis employing uncultured amniocytes might be a practicable way for prognostic evaluation of *PCDH19*-disorder in prenatal. The most shortcoming of this study is the limited case number and type; thus, clinicians should be cautious when applying these conclusions in clinical practice, while we still believe that our experiences will provide useful references for prenatal risk assessment of heterozygous X-linked deletions and laid a foundation for further systemic studies of prognostic valuation of XCI pattern for these cases.

## Data Availability

The raw data supporting the conclusion of this article will be made available by the authors, without undue reservation. The CMA and karyotype data analyzed in the study was obtained from Chongqing health center for women and children. According to the Regulations on Management of Human Genetic Resources in China, the domestic prenatal genetic data cannot be copied or uploaded. Requests to access these datasets should be directed to the prenatal diagnostic center.

## References

[B1] Berland S. Alme K. Brendehaug A. Houge G. Hovland R. (2011). PHF6 Deletions May Cause Borjeson-Forssman-Lehmann Syndrome in Females. Mol. Syndromol. 1 (6), 294–300. 10.1159/000330111 22190899 PMC3214959

[B2] Brown C. Robinson W. (2000). The Causes and Consequences of Random and Non-random X Chromosome Inactivation in Humans. Clin. Genet. 58 (5), 353–363. 10.1034/j.1399-0004.2000.580504.x 11140834

[B3] Crawford J. Lower K. M. Hennekam R. C. Van Esch H. Megarbane A. Lynch S. A. (2006). Mutation Screening in Borjeson-Forssman-Lehmann Syndrome: Identification of a Novel De Novo PHF6 Mutation in a Female Patient. J. Med. Genet. 43 (3), 238–243. 10.1136/jmg.2005.033084 15994862 PMC2563250

[B4] Di-Battista A. Meloni V. A. da Silva M. D. Moysés-Oliveira M. Melaragno M. I. (2016). Unusual X-Chromosome Inactivation Pattern in Patients with Xp11.23-p11.22 Duplication: Report and Review. Am. J. Med. Genet. 170 (12), 3271–3275. 10.1002/ajmg.a.37888 27605428

[B5] Dibbens L. M. Kneen R. Bayly M. A. Heron S. E. Arsov T. Damiano J. A. (2011). Recurrence Risk of Epilepsy and Mental Retardation in Females Due to Parental Mosaicism of PCDH19 Mutations. Neurology 76 (17), 1514–1519. 10.1212/WNL.0b013e318217e7b6 21519002

[B6] Dimova P. S. Kirov A. Todorova A. Todorov T. Mitev V. (2012). A Novel PCDH19 Mutation Inherited from an Unaffected Mother. Pediatr. Neurol. 46 (6), 397–400. 10.1016/j.pediatrneurol.2012.03.004 22633638

[B7] Disteche C. M. (1999). Escapees on the X Chromosome. Proc. Natl. Acad. Sci. 96 (25), 14180–14182. 10.1073/pnas.96.25.14180 10588671 PMC33938

[B8] Gendrel A.-V. Heard E. (2011). Fifty Years of X-Inactivation Research. Development 138 (23), 5049–5055. 10.1242/dev.068320 22069183

[B9] Ghi T. Sotiriadis A. Calda P. Da Silva Costa F. Raine-Fenning N. Alfirevic Z. (2016). ISUOG Practice Guidelines: Invasive Procedures for Prenatal Diagnosis. Ultrasound Obstet. Gynecol. 48 (2), 256–268. 10.1002/uog.15945 27485589

[B10] He W. B. Du J. Xie P. Y. Zhou S. Zhang Y. X. Lu G. X. (2019). X‐chromosome Inactivation Pattern of Amniocytes Predicts the Risk of Dystrophinopathy in Fetal Carriers of DMD Mutations. Prenatal Diagn. 39 (8), 603–608. 10.1002/pd.5473 31069818

[B11] Hoshina N. Johnson-Venkatesh E. M. Hoshina M. Umemori H. (2021). Female-specific Synaptic Dysfunction and Cognitive Impairment in a Mouse Model of PCDH19 Disorder. Science 372 (6539), eaaz3893. 10.1126/science.aaz3893 33859005 PMC9873198

[B12] Jahani-Asl A. Cheng C. Zhang C. Bonni A. (2016). Pathogenesis of Börjeson-Forssman-Lehmann Syndrome: Insights from PHF6 Function. Neurobiol. Dis. 96, 227–235. 10.1016/j.nbd.2016.09.011 27633282 PMC5102843

[B13] Jones J. R. (2014). Nonrandom X Chromosome Inactivation Detection. Curr. Protoc. Hum. Genet. 80, Unit 9 7. 10.1002/0471142905.hg0907s80 24510685

[B14] Leppig K. A. Disteche C. M. (2001). Ring X and Other Structural X Chromosome Abnormalities: X Inactivation and Phenotype. Semin. Reprod. Med. 19 (2), 147–158. 10.1055/s-2001-15395 11480912

[B15] Livak K. J. Schmittgen T. D. (2001). Analysis of Relative Gene Expression Data Using Real-Time Quantitative PCR and the 2−ΔΔCT Method. Methods 25 (4), 402–408. 10.1006/meth.2001.1262 11846609

[B16] Lyon M. F. (1961). Gene Action in the X-Chromosome of the Mouse (*Mus musculus* L.). Nature 190, 372–373. 10.1038/190372a0 13764598

[B17] Lyon M. (2002). X-chromosome Inactivation and Human Genetic Disease. Acta Paediatr. Suppl. 91 (439), 107–112. 10.1111/j.1651-2227.2002.tb03120.x 12572852

[B18] Machado F. B. Machado F. B. Faria M. A. Lovatel V. L. Alves da Silva A. F. Radic C. P. (2014). 5meCpG Epigenetic marks Neighboring a Primate-Conserved Core Promoter Short Tandem Repeat Indicate X-Chromosome Inactivation. PLoS One 9 (7), e103714. 10.1371/journal.pone.0103714 25078280 PMC4117532

[B19] Minks J. Robinson W. P. Brown C. J. (2008). A Skewed View of X Chromosome Inactivation. J. Clin. Invest. 118 (1), 20–23. 10.1172/JCI34470 18097476 PMC2147673

[B20] Ørstavik K. H. (2009). X Chromosome Inactivation in Clinical Practice. Hum. Genet. 126 (3), 363–373. 10.1007/s00439-009-0670-5 19396465

[B21] Puck J. M. Willard H. F. (1998). X Inactivation in Females with X-Linked Disease. N. Engl. J. Med. 338 (5), 325–328. 10.1056/NEJM199801293380611 9445416

[B22] Rastan S. (1982). Primary Non-random X-Inactivation Caused by Controlling Elements in the Mouse Demonstrated at the Cellular Level. Genet. Res. 40 (2), 139–147. 10.1017/s0016672300019017 7152255

[B23] Riggs E. R. Andersen E. F. Cherry A. M. Kantarci S. Kearney H. Patel A. (2020). Technical Standards for the Interpretation and Reporting of Constitutional Copy-Number Variants: a Joint Consensus Recommendation of the American College of Medical Genetics and Genomics (ACMG) and the Clinical Genome Resource (ClinGen). Genet. Med. 22 (2), 245–257. 10.1038/s41436-019-0686-8 31690835 PMC7313390

[B24] Samanta D. (2020). PCDH19-Related Epilepsy Syndrome: A Comprehensive Clinical Review. Pediatr. Neurol. 105, 3–9. 10.1016/j.pediatrneurol.2019.10.009 32057594

[B25] Santos-Rebouças C. B. Boy R. Vianna E. Q. Gonçalves A. P. Piergiorge R. M. Abdala B. B. (2020). Skewed X-Chromosome Inactivation and Compensatory Upregulation of Escape Genes Precludes Major Clinical Symptoms in a Female with a Large Xq Deletion. Front. Genet. 11, 101. 10.3389/fgene.2020.00101 32194616 PMC7064548

[B26] Schwartz C. E. (2013). Brenner's Encyclopedia of Genetics. Second Edition. Elsevier.

[B27] Terracciano A. Specchio N. Darra F. Sferra A. Bernardina B. D. Vigevano F. (2012). Somatic Mosaicism of PCDH19 Mutation in a Family with Low-Penetrance EFMR. Neurogenetics 13 (4), 341–345. 10.1007/s10048-012-0342-9 22949144

[B28] Tukiainen T. Villani A. C. Villani A.-C. Yen A. Rivas M. A. Marshall J. L. (2017). Landscape of X Chromosome Inactivation across Human Tissues. Nature 550 (7675), 244–248. 10.1038/nature24265 29022598 PMC5685192

[B29] Yin A.-h. Peng C.-f. Zhao X. Caughey B. A. Yang J.-x. Liu J. (2015). Noninvasive Detection of Fetal Subchromosomal Abnormalities by Semiconductor Sequencing of Maternal Plasma DNA. Proc. Natl. Acad. Sci. USA 112 (47), 14670–14675. 10.1073/pnas.1518151112 26554006 PMC4664371

[B30] Yuan H. Shangguan S. Li Z. Luo J. Su J. Yao R. (2021). CNV Profiles of Chinese Pediatric Patients with Developmental Disorders. Genet. Med. 23 (4), 669–678. 10.1038/s41436-020-01048-y 33402738

[B31] Zhang X. Fan Y. Liu X. Zhu M.-A. Sun Y. Yan H. (2019). A Novel Nonsense Mutation of PHF6 in a Female with Extended Phenotypes of Borjeson-Forssman-Lehmann Syndrome. J. Clin. Res. Pediatr. Endocrinol. 11 (4), 419–425. 10.4274/jcrpe.galenos.2019.2018.0220 30630810 PMC6878345

[B32] Zweier C. Rittinger O. Bader I. Berland S. Cole T. Degenhardt F. (2014). Females with De Novo Aberrations inPHF6: Clinical Overlap of Borjeson-Forssman-Lehmann with Coffin-Siris Syndrome. Am. J. Med. Genet. 166 (3), 290–301. 10.1002/ajmg.c.31408 25099957

