# Supplementary material for: X Chromosome Inactivation Pattern and Pregnancy Outcome of Female Carriers of Pathogenic Heterozygous X-Linked Deletions
Source: Front Genet. 2021 Dec 17;12:782629. doi: 10.3389/fgene.2021.782629 (PMC8719196; doi:10.3389/fgene.2021.782629)
Supplement: Supplementary file 1 [file DataSheet2.PDF]

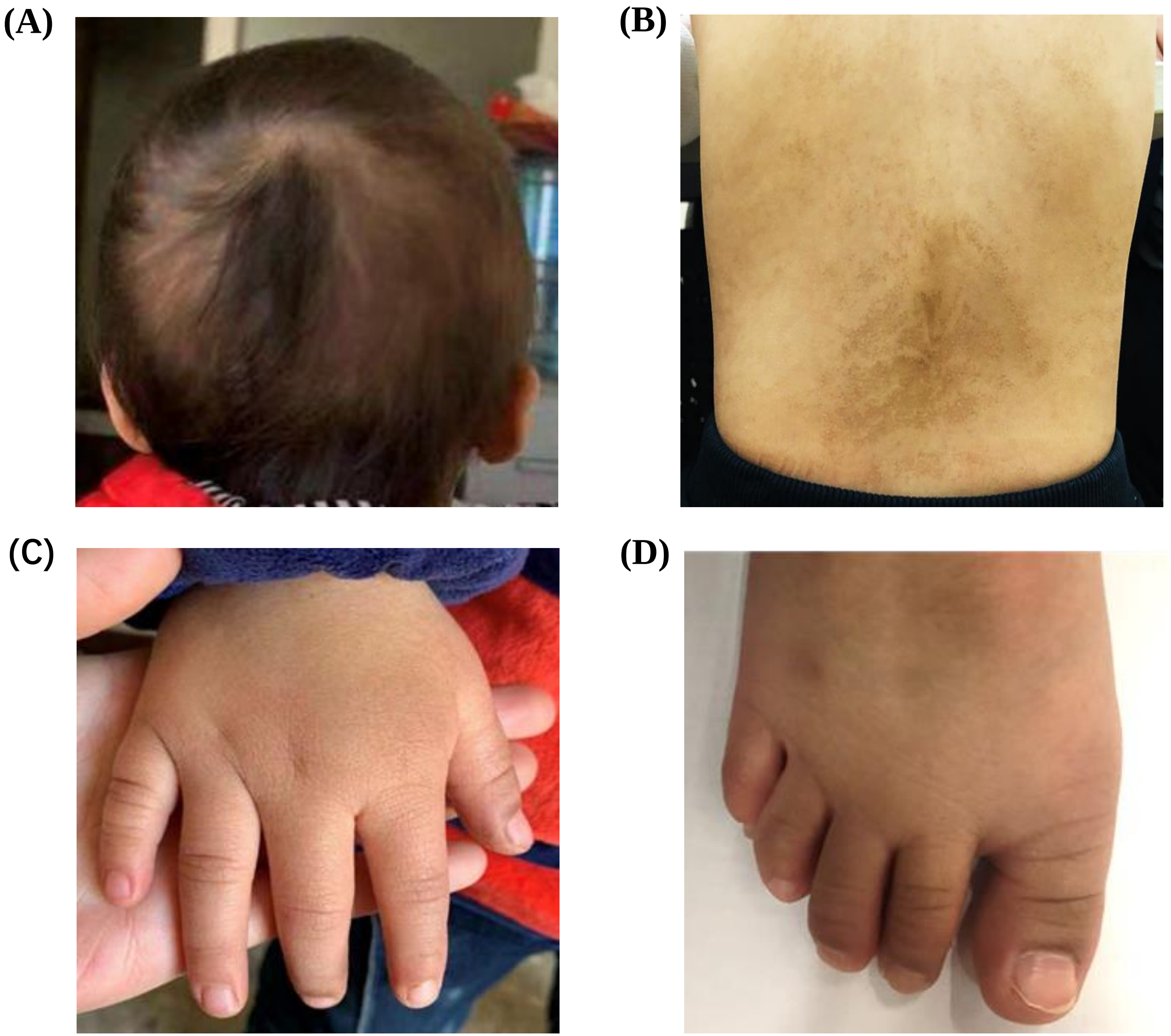

Supplementary Figure 2. Main physical features of the female patient with BFLS in pedigree 4. (A):Sparse hair; (B): Skin hyperpigmentation on the back; (C)&(D): Tapering and fifth curved fingers/toes.
