# Supplementary material for: X Chromosome Inactivation Pattern and Pregnancy Outcome of Female Carriers of Pathogenic Heterozygous X-Linked Deletions
Source: Front Genet. 2021 Dec 17;12:782629. doi: 10.3389/fgene.2021.782629 (PMC8719196; doi:10.3389/fgene.2021.782629)
Supplement: Supplementary file 2 [file Table1.DOCX]

Supplementary table Clinical data of four pedigrees

| **Pedigree** | **Family member** | **Genetic test** | **Result of test** |
| --- | --- | --- | --- |
| 1 | Fetus (Ⅲ-1) | NIPT | 15q11.2-15q13.1 deletion |
|  |  | CMA | arr[GRCh37] Xq21.33q22.1(96821302_100378384) x1 |
|  | Gravida (Ⅱ-2) | CMA | arr[GRCh37] Xq21.33q22.1(96821302_100378384) x1 |
|  |  | Q-PCR | 1 copy |
|  | Gravida’s husband (Ⅱ-3) | Karyotyping | 46,XY |
|  | Gravida’s father (Ⅰ-1) | Q-PCR | 1 copy |
|  | Gravida’s mother (Ⅰ-2) | Q-PCR | 1 copy |
| **2** | Fetus (Ⅳ-1) | NIPT | Xp11.23-Xp11.22 deletion |
|  |  | CMA | arr[GRCh37]  Xp11.23p11.22(47249368_50896523) x1 |
|  | Gravida (Ⅲ-3) | CMA | arr[GRCh37]  Xp11.23p11.22(47249368_50896523) x1 |
|  |  | Q-PCR | 1 copy of |
|  | Gravida’s husband (Ⅲ-4) | Karyotyping | 46,XY |
|  | Gravida’s father (Ⅱ-1) | Q-PCR | 1 copy |
|  | Gravida’s mother (Ⅱ-2) | Q-PCR | 2 copies |
|  | Gravida’s sister (Ⅲ-2) | Q-PCR | 2 copies |
|  | Gravida’s maternal aunt (Ⅱ-4) | Q-PCR | 2 copies |
| **3** | Fetus (Ⅲ-4) | NIPT | Xp22.33-Xp22.13 deletion |
|  |  | CMA | arr[GRCh37]  Xp22.33p22.13(178624_18404079) x1 |
|  | Gravida (Ⅱ-1) | Karyotyping | 46,X,del(X)(p22.1) |
|  | Gravida’s husband (Ⅱ-2) | Karyotyping | 46,XY |
|  | Gravida’s mother (Ⅰ-2) | Karyotyping | 46,X,del(X)(p22.1) |
| 4 | Proband (Ⅳ-1) | CMA | arr[GRCh37] Xq26.1q27.3(129511205_142742928)x1 |
|  |  | WES | Negative |
|  | Proband’s mother (Ⅲ-2) | CMA | arr[GRCh37] Xq26.1q27.3(129503423_142740132)x1 |
|  |  | WES | Negative |
|  | Proband’s father (Ⅲ-1) | Karyotyping | 46,XY |
|  |  | WES | Negative |
|  | Proband’s maternal grandfather  (Ⅱ-4) | Karyotyping | 46,XY |
|  | Proband’s maternal grandmother  (Ⅱ-3) | Karyotyping | 46,X,del(X)(q24q26) |
|  | Maternal grandmother’s sister 1  (Ⅱ-1) | Karyotyping | 45,X[8]/47,XXX[2],46,XX[51] |
|  | Maternal grandmother’s sister 2  (Ⅱ-2) | Karyotyping | 46,XX |
|  | Maternal grandmother’s sister 3  (Ⅱ-5) | Karyotyping | 46,XX |
|  | Maternal grandmother’s sister 4  (Ⅱ-6) | Karyotyping | 46,XX |
|  | Maternal grandmother’s sister 5  (Ⅱ-7) | Karyotyping | 46,XX |

The clinical information of family members who underwent genetic tests was summarized in this table. In pedigree 1,2,3, amniocentesis followed by CMA testing was performed for the risky indication of NIPT. In pedigree 4, CMA using peripheral blood cells was employed to make a postnatal diagnosis for the proband who presented with global developmental delay. For pedigree verification of the X-linked deletions found in probands, CMA and Q-PCR were employed to detect the deletion region below 5Mb, and karyotyping (G-band analysis) was used to detect the deletion region above 10Mb. Trio-WES was also conducted in pedigree four to preclude potential disease-related single nucleotide variants or indels, negative result means no pathogenic variant could well explain the patient’s phenotype.
