# Supplementary material for: X Chromosome Inactivation Pattern and Pregnancy Outcome of Female Carriers of Pathogenic Heterozygous X-Linked Deletions
Source: Front Genet. 2021 Dec 17;12:782629. doi: 10.3389/fgene.2021.782629 (PMC8719196; doi:10.3389/fgene.2021.782629)
Supplement: Supplementary file 3 [file DataSheet1.PDF]

(A)

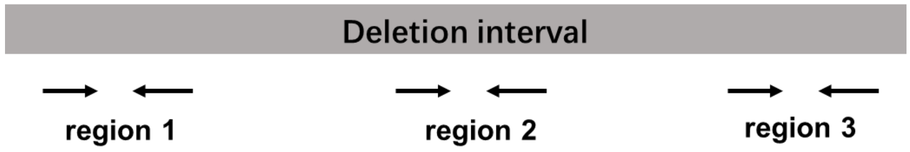
Deletion interval
region 1
region 2
region 3

(B)

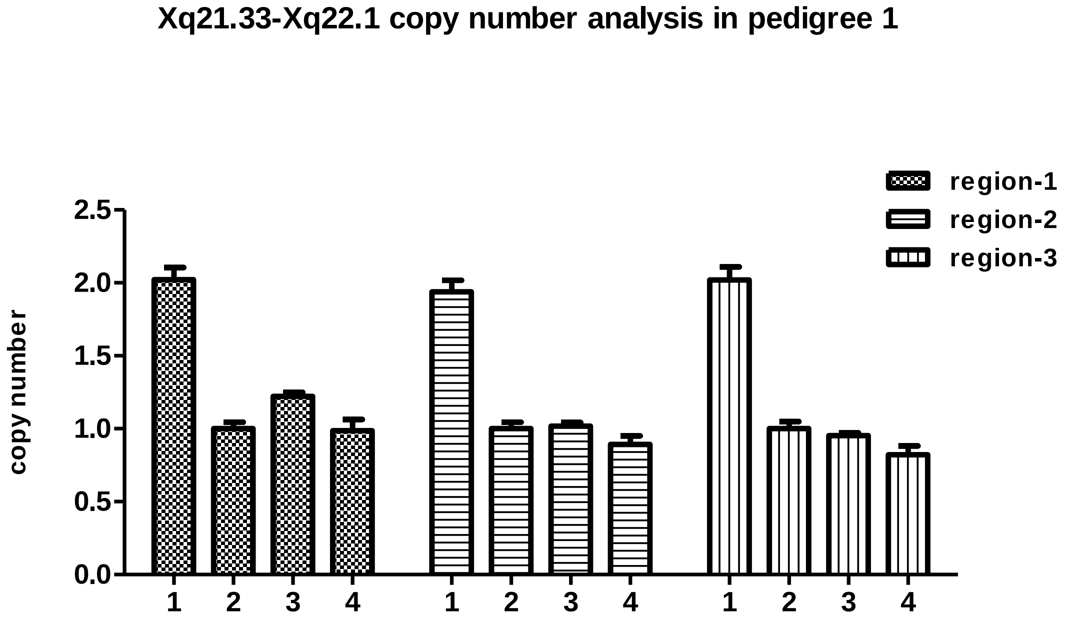
Xq21.33-Xq22.1 copy number analysis in pedigree 1
region-1
region-2
region-3
copy number
2.5
2.0
1.5
1.0
0.5
0.0
1 2 3 4 1 2 3 4 1 2 3 4

(C)

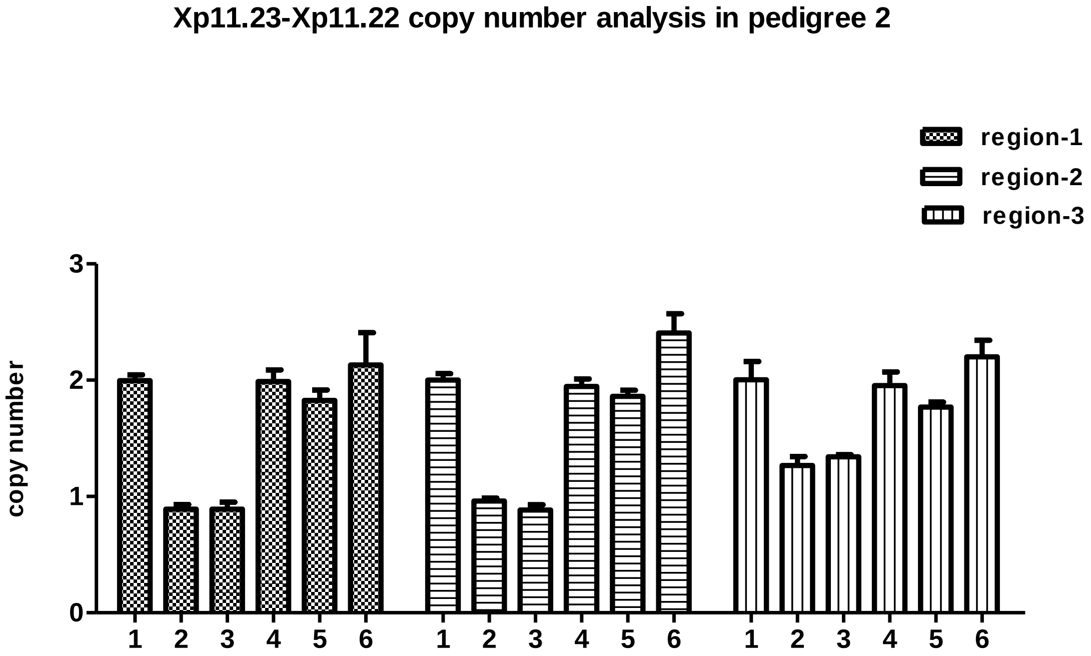
Xp11.23-Xp11.22 copy number analysis in pedigree 2
region-1
region-2
region-3
copy number
3
2
1
0
1 2 3 4 5 6 1 2 3 4 5 6 1 2 3 4 5 6

Supplementary Figure 1. Copy number variation with quantitative real-time PCR. (A): Schematic representation of the primer design for copy number assay. Three pairs of primer (indicated by black arrow) were designed to amplify the distinct areas of the deletion regions respectively (two of them cover the terminal coding regions and one covers the middle coding region of the deletion interval). (B): Estimation of Xq21.33-Xq22.1(96821302_100378384, hg19) copy number in pedigree 1. The number on X-axis represents sample number. 1: calibrator sample (normal female); 2: gravida's father (I-1); 3: gravida (II-2); 4: gravida's mother (I-2). Each sample bar represents the mean calculated copy number and error bars show the standard deviation for triplicate. Heterozygous deletions (one copy) were detected in the gravida and her mother. (C): Estimation of Xp11.23-Xp11.22(47249368_50896523, hg19) copy number in pedigree 2. The number on X-axis represents sample number. Each sample bar represents the mean calculated copy number and error bars show the standard deviation for triplicate. 1: calibrator sample (normal female); 2: gravida's father (II-1); 3: gravida (III-3); 4: gravida's sister (III-2); 5: gravida's mother (II-2); 6: gravida's maternal aunt (II-4). Heterozygous deletion (one copy) was detected in the gravida, other tested females contain two copies of this region.
